# Dysregulation in the Expression of Platelet Surface Receptors in Acute Coronary Syndrome Patients—Emphasis on P2Y12

**DOI:** 10.3390/biology11050644

**Published:** 2022-04-22

**Authors:** Rafał Szelenberger, Michał Seweryn Karbownik, Michał Kacprzak, Ewelina Synowiec, Sylwia Michlewska, Michał Bijak, Marzenna Zielińska, Alina Olender, Joanna Saluk-Bijak

**Affiliations:** 1 Department of General Biochemistry, Faculty of Biology and Environmental Protection, University of Lodz, 90-236 Lodz, Poland; joanna.saluk@biol.uni.lodz.pl; 2 Biohazard Prevention Centre, Faculty of Biology and Environmental Protection, University of Lodz, 90-236 Lodz, Poland; michal.bijak@biol.uni.lodz.pl; 3 Department of Pharmacology and Toxicology, Medical University of Lodz, 90-752 Lodz, Poland; michal.karbownik@umed.lodz.pl; 4 Department of Interventional Cardiology, Medical University of Lodz, 91-213 Lodz, Poland; michal.kacprzak@umed.lodz.pl (M.K.); marzenna.zielinska@umed.lodz.pl (M.Z.); 5 Laboratory of Medical Genetics, Faculty of Biology and Environmental Protection, University of Lodz, 90-236 Lodz, Poland; ewelina.synowiec@biol.uni.lodz.pl; 6 Laboratory of Microscopic Imaging and Specialized Biological Techniques, Faculty of Biology and Environmental Protection, University of Lodz, 90-237 Lodz, Poland; sylwia.michlewska@biol.uni.lodz.pl; 7 Chair and Department of Medical Microbiology, Medical University of Lublin, 20-093 Lublin, Poland; alina.olender@umlub.pl

**Keywords:** blood platelets, acute coronary syndrome, surface receptors, miRNA, P2Y12, GP IIb/IIIa

## Abstract

**Simple Summary:**

Acute Coronary Syndrome is a disease of the circulatory system characterized by the partial or complete blockage of coronary arteries. In thrombosis, a major role is played by platelets—the smallest, anucleate, morphotic elements in the bloodstream. Platelets are involved in the process of hemostasis, which ensures the continuity of the blood vessel by forming a clot that prevents blood loss. Unique structures of blood platelets ensure their high reactivity in the vascular microenvironment due to the interaction with many biologically active molecules, which is recognized by the surface receptors. The research carried out in the manuscript is aimed at detecting potential changes at the molecular level in platelet surface receptors that could constitute the potential importance of the occurrence of Acute Coronary Syndrome. The obtained results indicate that the P2Y12 receptor, which is the main target of antiplatelet therapy, is expressed more frequently among patients. In addition, we have shown that at the genetic level, quantitative changes also occur in the case of other receptors that are important in the activation of platelets. The following manuscript suggests the potential mechanisms responsible for the differences between patients and healthy donors due to a better understanding of the molecular causes of Acute Coronary Syndrome pathogenesis.

**Abstract:**

The pathological conditions caused by blood platelet activation constitute a fundamental core in the pathogenesis of Acute Coronary Syndrome (ACS). The hyperactivity of platelets in ACS is well-documented, but there is still little research into the molecular basis of phenotypic changes in platelet functionality. To expand the knowledge of this phenomenon, we analyzed the disturbances in the expression of several key platelet receptors and the aspect of regulating potential abnormalities. Platelet surface receptors are responsible for maintaining the hemostatic balance, platelet interaction with immune cells, and support of the coagulation cascade leading to occlusion of the vessel lumen. Due to their prominent role, platelet receptors constitute a major target in pharmacological treatment. Our work aimed to identify the molecular alteration of platelet surface receptors, which showed augmented mRNA expression of P2Y12, GP1BB, ITGA2B, and ITGB3 and increased protein concentrations of P2Y12 and GP IIb/IIIa in ACS. The upregulation of the P2Y12 level was also confirmed by confocal and cytometric visualization. Furthermore, we evaluated the expression of two microRNAs: miR-223-3p and miR-126-3p, which were suggested to regulate platelet P2Y12 expression. Results of our study present new insight into the molecular background of ACS.

## 1. Introduction

Acute Coronary Syndrome (ACS) describes the myocardial ischemic events arising from partial or complete blockage of coronary arteries. ACS can be divided into the three different clinical manifestations characterized by the occurrence of the various symptoms, including ST-segment elevation myocardial infarction (STEMI), non-ST-segment elevation myocardial infarction (NSTEMI), and unstable angina (UA) [1]. The major role in the pathogenesis of ACS is performed by blood platelets, the smallest morphotic elements that play a fundamental role in ensuring the hemostasis process. The physiological functioning of blood platelets is strictly controlled and begins with the adhesion to the injured vessel wall, which is accomplished with the formation of a blood clot that prevents blood loss. However, under certain pathological conditions, platelets may spontaneously form pathogenic aggregates, which constitute a fundamental factor in the development of cardiovascular diseases, including ACS [2]. Complex processes responsible for the platelet plug formation require a series of events including initiation, stimulation, and thrombus stabilization. All phases determine the dynamics of clot formation and are coordinated by a vast amount of platelet surface receptors [3]. The conditions prevailing in the vascular environment are extremely important for platelets. The surface-receptor-dependent adhesion process induces intracellular signal transduction, which leads to the activation of platelets, shape change, release of biologically active compounds from granules, and aggregation [4]. Activation of platelet pathways leads to recruitment of cells from the bloodstream; induces accumulation; enhances secondary aggregation induced by ADP; and provides overall interaction of platelets with leukocytes, endothelium, and coagulation factors [5]. Multitude platelet surface receptors ensure all necessary abilities of platelets to carry out hemostasis, thus emphasizing their important role in the proper functioning of these cells [3,4,5].

Structural and functional changes in the surface receptors of blood platelets may therefore play a pivotal role in the pathogenesis of ACS. Platelet hyperreactivity, the extension of blood procoagulative potential, development of the inflammatory processes, and increased interaction between platelet activators and their receptors constitute a complicated network of mutual relationships. One of the major targets for decreasing platelets reactivity by pharmacological blockade is the P2Y12 receptor. According to the novel guidelines of The European Society of Cardiology, the gold standard therapy for patients diagnosed with ACS include aspirin and a potent P2Y12 receptor inhibitor, with a recommendation for ticagrelor or prasugrel [6]. However, because of the various clinical presentations and characteristics of patients, the presence of comorbidities, comedication, and procedural aspects, the inhibitors of glycoprotein (GP) IIb/IIIa also constitute an effective antiplatelet therapy [6]. Besides the key role of the platelet surface receptors in the pathogenesis and prevention of ACS, studies related to their activity are largely reduced to testing new antiplatelet drugs. Our study aims to determine the molecular basis of the observed functional changes in blood platelets in ACS. The research hypothesis in this study assumes that a hyperreactivity of blood platelets in ACS may be caused by the altered expression of platelet surface receptors. In this study, we found that the expression on the mRNA level of major platelet surface receptors (P2Y12, ITGA2B, ITGB3, GP1BB) was significantly elevated in ACS patients. Furthermore, the concentrations of two main pharmacological targets, P2Y12 and subunit of GP IIb/IIIa, were significantly increased in the study group. Additionally, to evaluate the potential regulatory mechanism of overexpressed P2Y12 at the mRNA level, the expression levels of two human blood platelet microRNAs (miRNAs), miR-223-3p and -126-3p, were determined. Moreover, our study for the first time presents the cytometric and confocal visualization of altered expression of platelet P2Y12 receptor between ACS patients and controls, thus providing novel data that demonstrate observable characteristics that differentiate the study group from healthy donors. 

## 2. Materials and Methods

### 2.1. Chemicals 

Glucose, KCL, Thiourea, Urea, 2-[4-(2-hydroxyethyl)piperazin-1-yl]ethanesulfonic acid (HEPES), 3-[(3-cholamidopropyl)dimethylammonio]-1-propanesulfonate (CHAPS), Bovine Serum Albumin (BSA), Tris, and NH_4_HCO_3_ were obtained from Sigma-Aldrich (Saint Louis, MO, USA). NaCl, NaHCO_3_, Citric acid, Sodium Citrate, and NaH_2_PO_4_ were obtained from POCh (Gliwice, Poland). PBS tablets were obtained from Biosigma (Venice, Italy).

### 2.2. Blood Collection

Blood samples from the study group were taken from 35 patients of both sexes, aged 30–65 years old from the Department of Interventional Cardiology of the Medical University of Lodz with angiographically documented episodes of ACS. Blood was drawn from the patients immediately after the necessary examinations and treatments to ensure the patient’s safety. Patients received a one-time dose of antiplatelet drugs based on clinical characteristics such as age, sex, race, clinical picture, procedural aspects, administered drugs, and possible comorbidities. ACS patients registered in the study were under 65 years old; had normal kidney function; were not addicted to alcohol and narcotics; and in the medical history, had never been diagnosed with connective tissue disorders, hyperthyroidism, diabetes mellitus, and/or cancer. Furthermore, enrolled subjects had BMI < 35. Patients enrolled in the study had not been previously diagnosed with any cardiovascular diseases and did not receive any antiplatelet drugs before ACS occurred. The control group consisted of donors without any problems with the circulatory system. All volunteers that qualified for the study before inclusion were subjected to the following medical tests: morphology, creatinine, TSH (Thyroid-Stimulating Hormone), coagulation, CRP (C-Reactive Protein), IgG (Immunoglobulin G), and IgM titers, ALT (Alanine Transaminase), AST (Aspartate Transaminase), and levels of glucose; LDL (Low-Density Lipoprotein), HDL (High-Density Lipoprotein), cholesterol, and triglycerides. Donors enrolled in the study were free from any diseases and did not administer any medications at least 2 weeks before the blood draw. All clinical and demographical characteristics of patients and donors are included in Table 1. The study was approved by the Committee of the Ethics of Research in Human Experimentation at the University of Lodz with resolution number 23/KBNN-UŁ/I/2017. All enrolled patients and controls signed an informed consent form before entering the study. All procedures were performed according to the Helsinki Declaration for Human Research. 

### 2.3. Blood Platelet Isolation and Purification

The whole blood samples were collected by the S-Monovette^®^ CPDA1 system (Sarstedt, Numbrecht, Germany) and centrifuged (1200 rpm, 12 min, 37 °C). The top layer of platelet-rich plasma (PRP) was transported to fresh tubes. To avoid leukocyte and erythrocyte contamination, MACS^®^ magnetic cell separation was used. Obtained PRP was purified using superparamagnetic particles conjugated with specific antibodies: anti-CD45 and anti-CD235a (Miltenyi Biotech, Bergisch Gladbach, Germany). Further, PRP with antibodies was applied on the MS Column (Miltenyi Biotech, Bergisch Gladbach, Germany) and on the manual separator (Miltenyi Biotech, Bergisch Gladbach, Germany). Antibodies linked with leukocytes and erythrocytes were retained in the magnetic area of the MS Column and purified PRP flowed to a fresh tube. All steps were performed according to the manufacturer’s protocol, except for the wash buffer components, which were changed to minimize the possibility of platelet activation. For washing steps, a modified buffer containing PBS, 2-mM Citrate, and 0.5% BSA was used. Platelets were isolated from purified PRP by density centrifugation (1400 rpm, 15 min). Isolated platelets were washed 2 times with modified Tyrode’s Buffer (127 mM NaCl, 2.7 mM KCl, 0.5 mM NaH_2_PO_4_, 12 mM NaHCO_3_, 5 mM HEPES, 5.6 mM glucose, pH 7.4), and suspended in RNAlater (Thermo Fisher Scientific, Waltham, MA, USA) to stabilize mRNA. Samples were stored at −80 °C.

### 2.4. Total RNA Isolation and Synthesis of Complementary DNA

Isolation of total RNA from previously collected and purified blood platelet samples was performed using Isolate II RNA Mini Kit (Bioline, London, England). In the first step, RNAlater was removed from the sample by adding ice-cold PBS with a 1:1 ratio and centrifuged (5000 rpm, 5 min, 4 °C). Further steps were performed following the manufacturer’s protocol. Total RNA samples were treated with DNase I to digest potential DNA contamination. In the next step, total RNA was reverse transcribed to obtain cDNA with Maxima First Strand cDNA Synthesis Kit (Thermo Fisher Scientific, Waltham, MA, USA) according to the included protocol. Obtained cDNA was suspended in nuclease-free water and stored at −80 °C in the form of a cDNA library until use. To prepare a cDNA template for miRNA detection, TaqMan™ Advanced miRNA cDNA Synthesis Kit (Thermo Fisher Scientific, Waltham, MA, USA) was used. To the total RNA samples, cel-miR-39-3p (Thermo Fisher Scientific, Waltham, MA, USA) was added as an exogenous control. The final concentration of cel-miR-39-3p in each sample was 10 pM. All steps were performed according to the manufacturer’s protocol.

### 2.5. Isolation of Protein Fraction from Platelets

Isolated and purified platelet samples (obtained from 2 mL of PRP) were washed 3 times with modified Tyrode’s Buffer to avoid plasma contamination. The cell pellet was suspended in a lysis buffer (7 M urea, 2 M thiourea, 4% CHAPS, 30 mM Tris) and mixed until dissolved. Prepared samples were stored at −32 °C for subsequent analysis.

### 2.6. Gene Expression and miRNA Analysis by the Real-Time Quantitative PCR (RT-qPCR) Technique

For the following genes: P2Y12 (Hs01881698_s1)*,* ITGB3 (Hs01001469_m1)*,* ITGA2B (Hs00166246_m1)*,* GP1BB (Hs02579226_s1)*,* F2R (Hs05045041_s1), and 18S rRNA (Hs99999901_s1) expression analysis was performed using RT-qPCR with TaqMan Assays (Thermo Fisher Scientific, Waltham, Massachusetts, USA). As a reference gene, the expression of 18S rRNA was used. The RT-qPCR thermal cycling conditions were as follows: polymerase activation (95 °C, 10 min), 40 cycles of denaturation (95 °C, 15 s) and extension (60 °C, 1 min). For miRNA analysis, the following TaqMan™ Advanced miRNA Assays (Thermo Fisher Scientific, Waltham, MA, USA) were used: hsa-miR-223-3p (477983_mir), hsa-miR-126-3p (477887_mir), hsa-miR-191-5p (477952_mir), and cel-miR-39-3p (478293_mir). The RT-qPCR thermal cycling conditions were set according to the TaqMan™ Fast Advanced Master Mix (Thermo Fisher Scientific, Waltham, MA, USA) and were as follows: polymerase activation (95 °C, 20 s), 40 cycles of denaturation (95 °C, 3 s) and extension (60 °C, 30 s). As a reference miRNA, the expression of endogenous miR-191-5p and exogenous cel-miR-39-3p was used. Exogenous control was synthesized by Thermo Fisher Scientific with the following sequence (5′ to 3′) (RNA)-Phos-UCACCGGGUGUAAAUCAGCUUG. Data normalization was performed on the mean value of endo- and exogenous reference controls for the ΔCt calculation. For miRNA quantification, cDNA samples were diluted 10 times for analysis.

### 2.7. Determination of Protein Concentration Using ELISA Technique

Based on the results obtained from gene expression analysis, P2Y12, GPIIIa, and GPIb were selected for the determination of its concentration by ELISA technique (FineTest, Wuhan, China). All steps were performed according to the manufacturer’s protocol. Absorbance was measured at 450 nm and protein concentration was calculated from the standard curve.

### 2.8. Determination of P2Y12 Platelet Surface Expression by Flow Cytometry Method

Measurement of P2Y12 receptor was performed in whole blood samples from ACS patients and healthy controls using the flow cytometry method. In the first stage of sample preparation, erythrocytes were lysed by BD FACS Lysing Solution (Beckton Dickinson, San Diego, CA, USA). Further, samples were incubated for 30 min in the dark, at 24 °C, with specific murine monoclonal antibodies: BB515 Anti-Human CD61 (Beckton Dickinson, San Diego, CA, USA), PE Anti-Human P2Y12 (BioLegend, San Diego, CA, USA). Next, samples were fixed with 1% CellFix (Beckton Dickinson, San Diego, CA, USA), and centrifuged (6200 rpm, 12 min, 24 °C). After centrifugation, the supernatant was discarded and the obtained pellet was dissolved in 800 µL of 0.9% NaCl. Subsequently, samples were immediately analyzed using CUBE6 flow cytometry with CyView Software v.1.5.5.8 (Partec, Görlitz, Germany). During all performed measurements, the fluorescence of 15,000 events was recorded every time. Gates for used fluorochromes were set based on the fluorescence signal of unstained probes. FSC parameters were used to identify platelet microparticles (FSC below 10^2^) and platelet aggregates (FSC above 10^3^). Surface expression of the P2Y12 receptor on CD61-positive cells was assessed by the fluorescence intensity.

### 2.9. Visualization of P2Y12 Receptor via Confocal Microscopy

Visualization of the P2Y12 receptor level on the human blood platelets was performed via confocal microscopy. Due to maintaining platelets in a resting state, Sepharose 2B-BSA gel was used to isolate pure platelets without any potentially aggressive methods that could activate them before imaging. To avoid any possibility of false-positive or false-negative results, no inhibitory substances were added during platelet isolation. In the first stage, Sepharose 2B-BSA gel was washed to discard the ethanol layer and suspended in the modified Tyrode’s buffer. Next, the gel was packed in the column and PRP was put on the top of the column. The platelet fraction was collected in a fresh 2-mL tube. Further, 20 µL of suspended platelets were transferred to the fresh tube containing 6 µL of antihuman P2Y12 antibody conjugated with PE fluorochrome (BioLegend, San Diego, CA, USA) and 4 µL of antihuman CD61 antibody conjugated with PerCP-Cy5.5 (BD Biosciences, San Jose, CA, USA), and incubated in darkness for 30 min. Furthermore, 20 µL of 1% CellFix (Beckton Dickinson, San Diego, CA, USA) was added and incubated for 1 h at 37 °C. To reduce the background glow in the confocal microscope, samples were centrifuged (5000 rpm, 5 min, 24 °C) and the supernatant was discarded. Samples were suspended in 30 µL of PBS for analysis and deposited on glass slides with CyGEL™ (Biostatus, Leicestershire, UK) to immobilize platelets before imaging. For microscopic imaging, the confocal laser scanning microscopy platform TVS SP8 (Leica Microsystems, Wetzlar, Germany) with the objective 100×/1.40 (HC APO CS2, Leica Microsystems, Wetzlar, Germany) was used. Samples were imaged with the following wavelength values of excitation and emission: 489 and 500–550 nm for PE; 565 and 540–620 nm for PerCP-Cy5.5. Leica Application Suite X (LAS X, Leica Microsystems, Wetzlar, Germany) was used for cell imaging. Visualization of the P2Y12 receptor was performed in the Laboratory of Microscopic Imaging and Specialized Biological Techniques in the Faculty of Biology and Environmental Protection at the University of Lodz.

### 2.10. Statistical Analysis

mRNA and miRNA expressions were presented as –ΔCt, whereas protein concentrations were log-transformed to bring their distribution closer to normal. The difference in miRNA, mRNA expression, and protein concentration between ACS patients and healthy controls was evaluated with the use of Student’s *t*-test or, in case of unequal variance, Welch’s *t*-test. The correlation between miRNA, mRNA, and protein expression/concentration was tested with general linear modeling techniques. To assess the relevance of dimensionality reduction between the measurands of interest, Kaiser–Meyer–Olkin (KMO) statistics and the Bartlett’s χ2 test were used. The model to differentiate ACS patients from healthy controls was built with the use of multivariate logistic regression and was internally validated with a 10-fold cross-validation procedure. The model was further illustrated with the receiver operating characteristic (ROC) curve (data included in the Supplementary Material Table S1 and Figure S1). *p*-values below 0.05 were considered statistically significant. The analysis was performed using STATISTICA 13.3 Software (StatSoft; Tulsa, OK, USA).

## 3. Results

### 3.1. Gene Expression Analysis of Selected Platelet Surface Receptors

**Figure 1:**
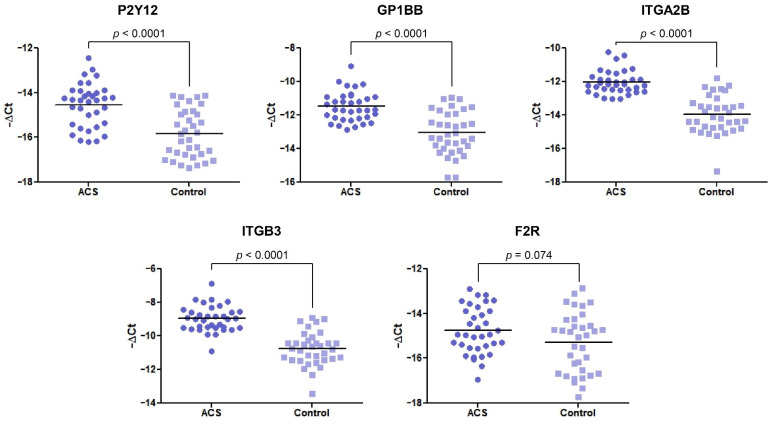
The mRNA expression levels of P2Y12, GP1BB, ITGA2B, ITGB3, and F2R in platelets of ACS patients compared with the control group. The relative expression of the selected genes was illustrated with −ΔCt value. 18S rRNA was used as a reference gene. Data are plotted as individual values with horizontal bars presenting the mean. The *p*-values reported above the graphs represent the results of the Student’s *t*-test, or, in cases of unequal variance, Welch’s *t*-test. F2R was not statistically significant with *p* = 0.074.

In our comparative analysis, we demonstrated a significant increase in the mRNA levels of P2Y12, GP1BB, ITGA2B, and ITGB3 genes (*p* < 0.0001) in the ACS group, compared with healthy volunteers. For the statistically significant results, alterations are presented as a fold change with 95% CI. For the abovementioned genes, the expression was a 2.44-fold (1.73–3.42), 2.93-fold (2.04–4.22), 3.77-fold (2.77–5.14), and 3.43-fold (2.56–4.59) increase in the ACS group compared with healthy donors, respectively (Figure 1). The expression at the mRNA level of F2R was not statistically significant; however, the upregulation was shifted towards the ACS group.

### 3.2. Determination of Protein Concentration by ELISA

Based on the results obtained from the expression analysis of genes encoding platelet surface receptors, we selected 3 proteins—P2Y12, GPIIIa, and GPIb—for concentration measurement in the study and control group. We decided to determine the concentration of only one GPIIb/IIIa subunit. The GPIIIa remains in molar equilibrium with the GPIIb subunit and its amount is the determinant of the creation of functional GPIIb/IIIa. 

**Figure 2:**
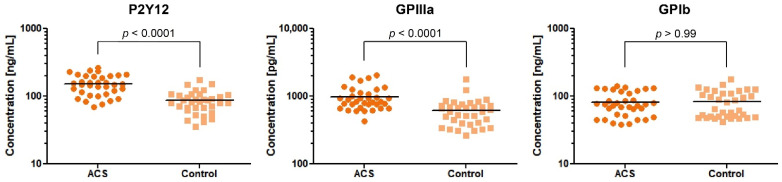
Levels of P2Y12, GPIIIa, and GPIb proteins in blood platelet lysates from ACS patients compared with the control group. Data are plotted as individual values with horizontal bars presenting the mean concentration. The *p*-values reported above the graphs represent the results of *t*-tests following log-transformation.

Our comparative analysis showed an increased concentration of P2Y12 and GPIIIa in blood platelet lysates of ACS patients in comparison with healthy donors, with fold-changes (95%CI) = 1.78 (1.49–2.11) and 1.61 (1.34–1.95), respectively. The median value (with 25% and 75% percentile) of concentration for P2Y12 were 149 ng/mL (108.1–200.9) in ACS patients compared with 82.66 ng/mL (67.20–105.3) in the control group (*p* < 0.0001). The concentration of GPIIIa in ACS patients was 843.5 pg/mL (676.8–1134) compared with 592.6 pg/mL (403.3–732.7) in the control group (*p* < 0.0001). The concentration of GPIb did not show any significant differences between studied groups (ACS patients, 75.29 ng/mL (51.09–117.3); control group, 65.46 ng/mL (49.75–111.1); *p* > 0.99) (Figure 2). Despite the significant results of the two major platelet receptors, our further attention was focused on the P2Y12 receptor due to its primary role in the antiplatelet therapy assessed by actual ESC guidelines [6].

### 3.3. Platelet Surface Expression of P2Y12 by Flow Cytometry

**Figure 3:**
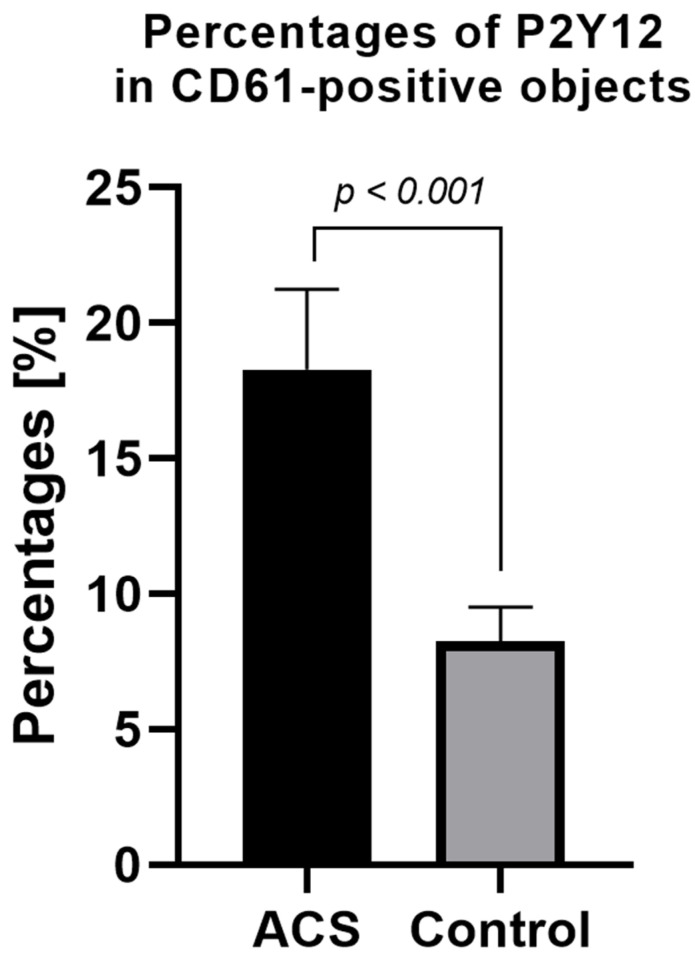
Platelet surface P2Y12 expression measured by flow cytometry method using PE Anti-Human P2Y12. Results presented in the figure show the percentage of P2Y12-positive targets in the population of CD61-positive subjects. Data are presented as a column bar graph with mean ± SD.

The expression level of surface P2Y12 in nonstimulated samples from ACS patients was significantly higher compared with the control group. The mean percentage of P2Y12 in CD61-positive, nonstimulated objects for the ACS group was approximately 18% (±2.95) vs. 8% (±1.26) in the control group (Figure 3). 

### 3.4. Visualization of Blood Platelets P2Y12 Receptor

**Figure 4:**
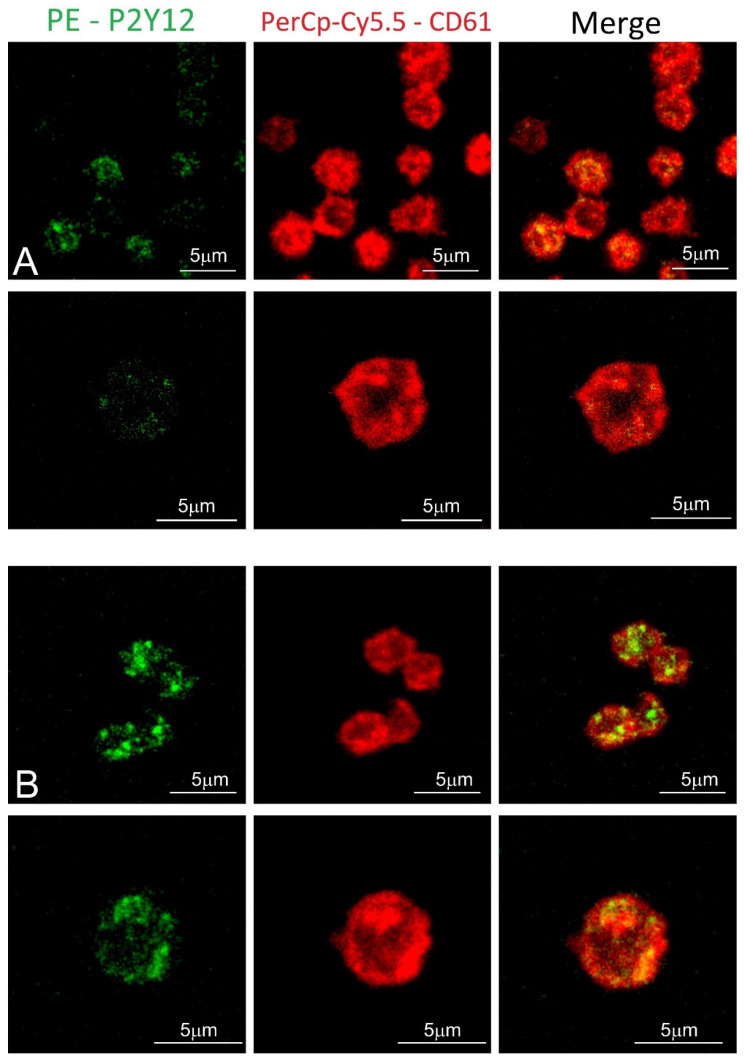
Confocal microscopy of fixed platelets. Samples were labeled with the platelet marker CD61-PerCP-Cy5.5 and with P2Y12-PE. Control and study platelets were deposited on the glass slides with CyGEL™. (**A**) blood platelets obtained from control donor; (**B**) blood platelets obtained from ACS patient.

Blood platelet samples from ACS patients and the control group were also analyzed by confocal microscopy using fluorescent-labeled antibodies. In the obtained images, the stronger fluorochrome emission for P2Y12 can be observed in ACS patients (Figure 4). Furthermore, receptors showed the tendency to be formed in clusters on the surface of blood platelets. 

### 3.5. Elevated Expression of miR-223-3p and miR-126-3p in Blood Platelets of ACS Patients

**Figure 5:**
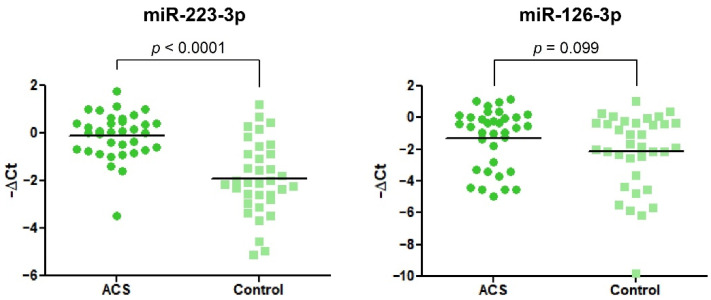
The relative expression of miR-223-3p and miR-126-3p in blood platelets of the study and control group. Data were calculated using a −ΔCt value with the means of both miR-191-3p and cel-miR-39-3p as reference miRNAs. Data are plotted as individual values with horizontal bars presenting the mean. The *p*-values reported above the graphs represent the results of Student’s *t*-tests or, in cases of unequal variance, Welch’s *t*-test.

Data obtained from the RT-qPCR showed significant alteration in the expression of miR-223-3p. We found that miR-223-3p was 3.48-fold (2.28–5.33) increased in ACS patients vs. healthy volunteers. In the case of miR-126-3p, we did not observe any statistical differences (Figure 5).

### 3.6. Protein-mRNA-miRNA Correlation in ACS Patients and Healthy Controls

The analysis of correlations between the statistically significant upregulated expressions of miR-223-3p, P2Y12 mRNA, and P2Y12 proteins was performed using a general linear modeling technique by adjusting for the group (ACS patient vs. healthy control), to account for its possibly confounding effect on the correlation. In this analysis, none of the pairs of measurands were significantly correlated: β = 0.05 (95%CI −0.20 to 0.30), *p* = 0.68 for miR-223 and P2Y12 mRNA pair; β = −0.05 (95%CI −0.28 to 0.18), *p* = 0.66 for miR-223 and P2Y12 protein pair; β = 0.10 (95%CI −0.13 to 0.32), *p* = 0.38 for P2Y12 mRNA and P2Y12 protein pair. Further, the group did not significantly differentiate the extent of any of these correlations as assessed in the interaction models: *p* = 0.50, *p* = 0.48, and *p* = 0.92 for miR-223 and P2Y12 pair, miR-223 and P2Y12 pair, and P2YR12 and P2Y12 pair, respectively. As a result, no dimensionality reduction between the tested measurands was warranted, as further confirmed by the KMO statistics of 0.490 and the Bartlett’s χ2 test result: χ2(3) = 0.87, *p* = 0.83. On the other hand, miR-223, P2Y12, and P2Y12 proteins could be validly included in the model construction to differentiate ACS patients from healthy controls.

## 4. Discussion

Human blood platelets are small, anucleate cells with a discoidal shape that freely circulate in blood vessels [7]. Their ultrastructure provides unique functional and behavioral properties. As a fragment of larger, precursor cells–megakaryocytes, blood platelets are surrounded by a phospholipid bilayer in which a massive number of surface receptors are anchored. The arrangement of phospholipids present in the inner layer is asymmetrical, thus providing the stability of the cell membrane. Surface receptors are mainly responsible for the activation of blood platelets on various pathways and for triggering the release of bioactive molecules contained in their α- and dense granules. Unleashed factors maintain not only the coagulation process but also inflammation, atherosclerosis, angiogenesis, tumorigenesis, bacterial interaction, and wound repair [4,8]. Due to the increased state of knowledge, suggesting that platelet dysfunction and related genetic disorders can have a decisive importance in ischemic events, we carried out a comparative analysis of gene expression and protein concentration of blood platelet surface receptors.

In the first stage, the expression of the selected platelet surface receptors on the mRNA level was evaluated. Results from RT-qPCR showed that P2Y12, GP1BB, ITGA2B, and ITGB3 mRNA transcripts were significantly upregulated in ACS patients (*p* < 0.0001) presenting 2.44-fold, 2.93-fold, 3.77-fold, and 3.43-fold elevation, respectively. Besides the fact that individual values and medians indicated the growing tendency in the ACS group, the statistical analysis showed no differences in the relative expression of F2R between studied groups (Figure 1). Furthermore, for receptors showing an increase in relative expression, the measurement of P2Y12, GPIb, and GPIIIa concentration in lysates obtained from blood platelets was performed. Results obtained from ELISA tests showed significant upregulation in the concentration of P2Y12 (*p* < 0.0001) and GPIIIa (*p* < 0.0001) (Figure 2). The augmented expression and concentration of GPIIIa may be associated with the increased synthesis of both GPIIb and GPIIIa subunits in blood platelets. Despite the lack of nuclei, de novo biosynthesis of proteins in human platelets has been confirmed in several studies [9,10,11,12]. Kieffer et al. [13], using radioactive labels, showed that circulating platelets are able to synthesize major membrane glycoproteins, including GPIIb and IIIa. The increased generation and presence of GP IIb/IIIa receptors may have a great impact on the hyperresponsiveness of blood platelets in ACS. In a study conducted by Yakushkin et al., the increased expression of GP IIb/IIIa was associated with a higher aggregation response [14], emphasizing that excessive exhibition of GP IIb/IIIa receptors for fibrinogen is an important factor of exaggerated aggregation of blood platelets that intensifies the process of thrombosis. The screening analysis of GP- IIb/IIIa and its subunits concentration could help in identifying a human predisposition to developing thromboembolic complications.

Further analysis performed in our study was focused on the P2Y12 receptor, especially because of its very important role in the pharmacotherapy of ACS patients. P2Y12 is a purinergic receptor for ADP that belongs to G-protein-coupled receptors (GPCRs) family. Its major role in human platelets is to inhibit the adenylyl cyclase, which is a key feature in the enhancement of platelet activation and stabilization of forming aggregates [15]. In a study conducted by Hu et al., the expression of human platelet P2Y12 on the mRNA and protein level was 4-fold higher in Type 2 Diabetes Mellitus (T2DM) than in healthy donors [16]. Obtained results were confirmed in the rats model that developed T2DM spontaneously in comparison with wild, Wistar rats. Furthermore, the authors did not observe statistically significant differences in F2R mRNA level either [16]. The statistically significant upregulation of mRNA and protein levels of P2Y12 were also observed in patients with secondary-progressive multiple sclerosis, which presents an increased risk of ischemic events such as stroke or myocardial infarction [17]. Unfortunately, there is a lack of studies presenting differences in protein concentration or gene expression of platelet P2Y12 in cardiovascular diseases. The possible explanation for the increased mRNAs expression may be associated with the role of splicing in blood platelets. Studies show that the transformation of blood platelets from the quiescent to the active state may stimulate the processing of the pre-mRNAs found in platelets into mature mRNAs via the functional spliceosome [18,19]. This phenomenon could explain the increased expression of mRNAs and proteins in the ACS group. However, as demonstrated by Nassa et al., in the case of P2Y12 receptor and GP IIb/IIIa subunits, there are no differences in expression depending on the state of the platelets (quiescent or activated), which may suggest that splicing does not participate in the mechanism of P2Y12 overexpression [20]. However, the lack of experimental data confirming the assumed hypothesis should be considered a limitation in our manuscript, and the explanation of this mechanism requires further research.

To confirm the augmented surface expression of P2Y12 in resting platelets, we decided to use confocal microscopy visualization. In the obtained images, stronger emission of P2Y12 PE fluorochrome can be observed in the ACS group. Interestingly, results showed that P2Y12 in the ACS group is more frequently observed in large clusters than being distracted in the cell membrane (Figure 4); however, the understanding of this phenomenon remains unclear. A possible explanation may be associated with the protein synthesis capacity of platelets, especially in the presence of an increased amount of transcripts; nonetheless, it needs further, detailed analysis. Results obtained from confocal microscopy were also confirmed in the flow cytometry, which, on the basis of fluorescent intensities, showed a significant increase in the surface expression of P2Y12 in ACS patients (Figure 3). Our data for the first time demonstrate the alteration in the expression of platelet surface receptors on the mRNA and protein level in patients with ACS compared with healthy controls.

To evaluate the potential mechanism associated with an increased quantity of mRNA transcripts for P2Y12, we decided to measure the expression of two miRNAs (miR-223-3p and miR-126-3p), which were shown to be associated with the P2Y12 receptor. Both miR-223 and -126 are some of the most abundant miRNAs stored in blood platelets [21]. In a study conducted by Landry et al., miR-223 was shown to regulate the expression of P2Y12 in the megakaryocytes, thus suggesting a concept in which miR-223 may regulate the expression of P2Y12 in blood platelets [22]. Our results showed that miR-223-3p was significantly upregulated in ACS patients compared with healthy controls (Figure 5). Similar results were shown in a study conducted by Li et al., where the level of platelet miR-223 was significantly elevated in STEMI patients compared with healthy controls [23]. Furthermore, Hromadka et al. demonstrated that the level of miR-223 in whole blood samples of Acute Myocardial Infarction (AMI) patients was significantly elevated [24]. Upregulation of miR-223 was also shown in the serum of AMI patients compared with healthy donors [25]. These findings are important due to the fact that miR-223 was shown to be exclusive for blood platelets [26]. However, there are also studies staying contrary to our and the abovementioned results. In studies conducted by Shi et al. and Zhang et al., the level of platelet and serum miR-223-3p was shown to be significantly decreased in post-myocardial infarction patients that received clopidogrel and presented a low response to used medicament, suggesting the potential role of miR-223 as a biomarker for pharmacotherapy monitoring [27,28]. These findings suggest that downregulation of miR-223 may be associated with the reduced post-transcriptional regulation of P2Y12 expression or be involved in the mechanisms of clopidogrel resistivity. In contrast, Leireseder et al. showed that miR-223-deficient mice did not differ in terms of platelet number, lifespan, volume, and level of platelet surface receptors from wild-type mice, suggesting that miR-223 did not affect the functionality of P2Y12 and is not associated with the ADP-induced aggregation. However, their theory may be controversial since bioinformatic analysis did not show a specific binding site for miR-223 in mice platelets [29]. Unfortunately, there is no clear explanation of simultaneous overexpression of P2Y12 and miR-223. Statistical analysis showed no correlation between expression of miR-223-3p, P2Y12 mRNA transcript, and P2Y12 protein concentration, which may suggest that miR-223-3p is not the key regulator of P2Y12 expression and finding the possible molecular mechanisms of action requires further detailed studies. However, a possible explanation for the parallel increase in the expression of miR-223-3p and P2Y12 may be the organism’s reaction to the appearance of a pathological condition, which, in order to restore homeostasis and reduce the expression of an excessively stimulated platelet receptor, increases the concentration of associated miRNA. Furthermore, blood samples of patients enrolled in our study were collected relatively fast after ACS incidence, where the effect of antiplatelet therapy may not be reflected on the platelet miRNA profile. In studies presenting downregulation of miR-223 in blood platelets, patients received antiplatelet agents from 1 to 5 days before blood collection, which might trigger significant changes at the molecular level of platelets and megakaryocytes. Furthermore, the results demonstrated by Shi et al. and Zhang et al. were not compared with healthy controls [27,28].

Our study also showed no significant alteration in the level of platelet miR-126-3p in ACS patients compared with healthy volunteers (Figure 5). In the current state of the art, there is great controversy regarding miR-126-3p expression. Several studies showed that circulating miR-126 is downregulated in patients with cardiovascular diseases in comparison with control subjects [30,31,32]. Liu et al. showed that the expression of plasma miR-126 is decreased in ACS patients and its level is associated with platelet reactivity during clopidogrel treatment [33]. On the other hand, Zampetaki et al. showed that the level of miR-126 was positively correlated with the incident of myocardial infarction [26]. Furthermore, Kazimierczyk et al. demonstrated that AMI patients with percutaneous coronary intervention also had elevated levels of circulating miR-126 [34]. The potential mechanism of miR-126 and P2Y12 receptor association was studied by Kaudewitz et al., where the application of antagomir-126-3p in a mice model caused the reduced expression of P2Y12 [35]. In a physiological aspect of this model, a reduced amount of miR-126-3p will be associated with the reduced expression of P2Y12. Taking into consideration the opposite direction of alteration in our study (despite the lack of statistical significance), increased expression of miR-126-3p could be linked with increased expression of P2Y12 at the mRNA level, which was shown in our study. In a study conducted by Garcia et al., the functionality of miR-126-3p was assessed on a platelet-like structure (PLS) derived from human hematopoietic stem cells. Results showed that PLS transfected with miR-126-3p had a 30% increased expression of P-selectin after thrombin stimulation. Furthermore, overexpression of miR-126-3p resulted in the downregulation of PLXBN2 expression, thus affecting RhoGTPase activity and actin dynamics regulation, demonstrating that overexpression of miR-126-3p may be associated with the increased reactivity of blood platelets [36]. Discrepancies in the results of cited studies may arise from the time of miRNAs expression measurements and time of blood sample collection after the ACS incident. Results mainly indicate that the downregulation of miR-126 expression is caused by antiplatelet therapy. Furthermore, there is a lack of studies that measure the level of miR-126-3p in blood platelets of patients with cardiovascular diseases. Moreover, it is suggested that miR-126 mainly originates in endothelial cells [37]; thus, its level in serum may not reflect the augmented expression in blood platelets. Altogether, miR-126-3p seems to be not very specific for differentiating ACS patients from healthy controls, and its possible role as a biomarker should be tested on the larger population.

Our study aimed to indicate the important role of the molecular basis underlying the observed functional changes in platelet surface receptors, especially P2Y12, in the pathogenesis of myocardial ischemia, with a possible finding of the potential biomarker. In a vast number of studies, the role of platelet surface receptors is examined from the perspective of novel antiplatelet drug introduction in clinical usage. However, there is a gap in molecular studies of changes in surface receptors, which may lead to the development of thrombosis. The possible hypothesis that could explain the hyperreactivity of platelets associated with the augmented expression of the P2Y12 receptor concerns the constitutive activity of GPCRs. The two-state model establishes that GPCRs existing in the active or inactive state remain in equilibrium. However, in particular conditions, the effector system and G-protein activity may be upregulated and cause spontaneous isomerization of GPCRs, thus changing from the inactive to the active state, in the absence of agonists [38]. In a study conducted by Zhang et al., transgenic mice expressing constitutively active chimeric P2Y12 showed an increased reactivity of blood platelets, shortened bleeding time, faster and more stable formation of thrombus, decreased level of platelet cyclic adenosine monophosphate (cAMP), and constitutive phosphorylation of Akt without agonists [39]. The constitutive activity of GPCRs with the simultaneous elevation of P2Y12 concentration may be the main factor of hyperreactivity of blood platelets in ACS patients, and early detection of this disorder could help in the identification of human predisposition to ischemic events.

## 5. Conclusions

To conclude, our manuscript presents an attempt to determine the molecular alterations of platelet surface receptors in ACS patients compared with healthy controls, by assessing changes in the expression level of transcripts, protein concentrations, as well as visualizing the arrangement of the platelet surface receptor P2Y12 by confocal microscopy. RTq-PCR analysis showed that mRNAs of P2Y12, GP1BB, ITGA2B, and ITGB3 were overexpressed in the ACS group, such as miR-223-3p. Furthermore, augmented protein concentration from blood platelet lysates was also found for P2Y12 and GPIIIa in ACS patients. What is more, confocal microscopy and flow cytometry show an elevated surface expression of P2Y12 in blood platelets of ACS patients in comparison with healthy donors. The results of our study may suggest the possible role of P2Y12 overexpression in the increased activity of blood platelets and in the pathogenesis of ACS; however, to fully understand this complex association between the overstimulated platelets and the level of their surface receptors, further molecular studies on larger populations are required.

## Figures and Tables

**Table 1:** Sociodemographic, anthropometric, and blood biochemical characteristics of ACS patients and healthy controls.

Parameter	ACS (*n* = 35)	Control (*n* = 35)	Reference Range	
	Median (1st–3rd Quartiles) or Number (Frequency)			*p*-Value
Sociodemographic and Anthropometric Characteristics				
Age (years)	50 (45–61)	48 (41–57)	-	0.545
Sex (male)	29 (83%)	28 (80%)	-	0.999
BMI (kg/m^2^)	30 (27–33)	29 (28–32)	<35	0.876
Blood biochemical characteristics				
Leukocytes (10^3^/µL)	8.60 (7.10–9.80)	5.98 (4.83–7.98)	4–11	<0.001
Erythrocytes (10^6^/µL)	4.47 (4.25–4.95)	5.07 (4.61–5.30)	4.2–6.1	0.0064
Blood platelets (10^3^/µL)	260 (202–287)	249 (208–292)	150–400	0.946
Glucose (mmol/L)	6.00 (5.31–6.35)	4.99 (4.76–5.57)	4.1–5.5	<0.001
Creatinine (µmol/L)	81.0 (72.8–90.0)	76.0 (69.9–87.5)	64–104	0.240
GFR (mL/min/1.73 m^2^)	96.7 (81.0–104.3)	91.5 (81.7–103.1)	>60	
AST (U/I)	34 (26–38)	19 (17–25)	<50	<0.001
ALT (U/I)	29 (21–42)	22 (22–38)	<50	0.120
Total cholesterol (mmol/L)	5.12 (4.36–5.96)	4.93 (4.37–5.42)	3–5	0.296
LDL (mmol/L)	3.07 (2.57–4.20)	2.84 (2.35–3.36)	-	0.085
HDL (mmol/L)	1.15 (1.02–1.33)	1.29 (1.12–1.67)	>1	0.009
Triglycerides (mmol/L)	1.67 (1.01–2.78)	1.24 (0.97–1.79)	<1.7	0.015
TSH (μIU/mL)	1.71 (1.16–2.53)	1.93 (1.31–2.61)	0.27–4.20	0.463

Clinical parameters are presented as a median and 1st–3rd quartile of 25th–75th percentile. Abbreviations: ALT—alanine transaminase; AST—aspartate transaminase; BMI—body mass index; GFR—glomerular filtration rate; HDL—high-density lipoprotein; LDL—low-density lipoprotein; TSH—thyroid-stimulating hormone.

## Data Availability

All data obtained from this study are included in the manuscript.

## Supplementary Materials

The following supporting information can be downloaded at: https://www.mdpi.com/article/10.3390/biology11050644/s1, Figure S1: Receiver operating characteristic curve for the multivariate logistic regression model differentiating ACS patients from healthy controls; Table S1: Characteristics of the logistic regression model differentiating ACS patients from healthy controls based on miR-223-3p as well as mRNA and protein levels of P2Y12.
