# Supplementary material for: Dysregulation in the Expression of Platelet Surface Receptors in Acute Coronary Syndrome Patients—Emphasis on P2Y12
Source: Biology (Basel). 2022 Apr 22;11(5):644. doi: 10.3390/biology11050644 (PMC9138357; doi:10.3390/biology11050644)
Supplement: Supplementary file 1 [file biology-11-00644-s001.zip › biology-1664707-supplementary/Supplementary Material S1.pdf]

biology MDPI

Supplementary Material S1: Biomarker modeling for differentiating ACS patients from healthy controls

As platelets of ACS patients were found to overexpress miR-223-3p as well as P2Y12 mRNA and P2Y12 protein, and the three measurands were negligibly correlated between each other, all of them could be included as predictors in the model differentiating ACS patients from healthy controls. Such a multivariate logistic model was constructed and internally validated with 10-fold cross-validation. The model appeared accurate and not prone to overfitting as it presented favorable goodness-of-fit statistics and performed well even in the validation set reaching high sensitivity with satisfactory specificity (Table S1, Figure S1). The obtained model reached 97% of sensitivity and 74% of specificity to differentiate ACS patients from donors.

Table S1. Characteristics of the logistic regression model differentiating ACS patients from healthy controls based on miR-223-3p as well as mRNA and protein levels of P2Y12. Likelihood-ratio $\chi2(3) = 59.65$, $p < 0.0001$. Hosmer-Lemeshow goodness-of-fit $\chi2(8) = 2.47$, $p = 0.96$**.**

**Predictor**	**Wald χ2**	Odds ratio [a]		*P*-value
		Point estimate	95% CI	
**miR-223-3p**	**9.69**	3.52	1.59–7.78	0.0019
P2Y12 mRNA	4.91	3.08	1.14–8.31	0.027
P2Y12 protein	10.44	16.85	3.04–93.53	0.0012

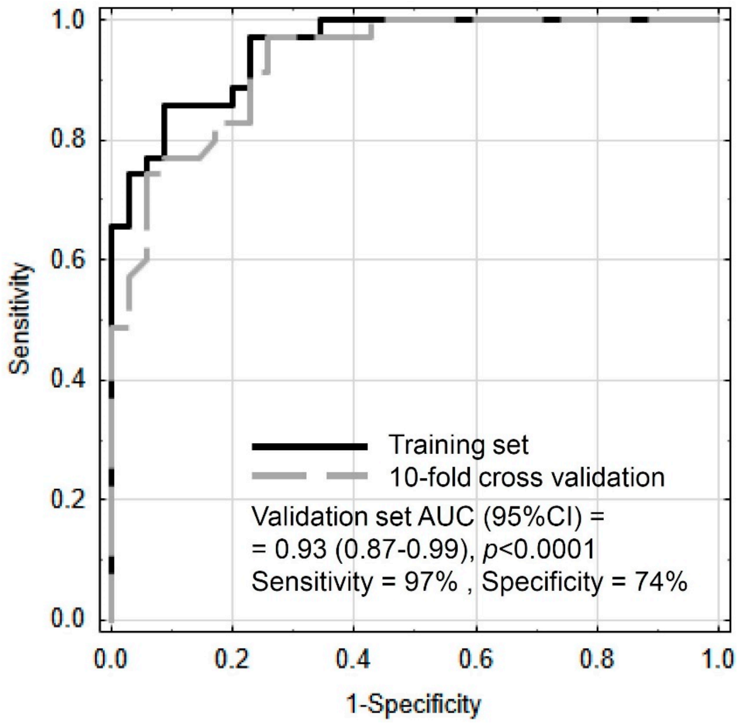

Figure S1. Receiver operating characteristic curve for the multivariate logistic regression model differentiating ACS patients from healthy controls. The model was based on protein and mRNA levels of P2Y12 as well as miR-223-3p. The sensitivity and specificity of the model were proposed based on the maximization of Youden's index. Model internal validation was performed with a 10-fold cross-validation technique.
